# 

**Published:** 1914-11-28

**Authors:** 


### CONTENTS.

PAGE

**The Wounded, the Women of England, and the Voluntary Hospitals** ... ... ... 185

**The State Control of Research** ... ... ... 186

**Hospital and Institutional News ...** ... ... 187

Scouts Help the Boot Fund—Patient's Attack on a Medical Superintendent—Soldiers' Stay in Hospital: Official Instructions—"Canvassing Will be Considered a Disqualification"—A Double Loss to the Seamen's Hospital—The Ommanney Family and the Seamen's Hospital—Belgian Medical Students in London—Medical Treatment of Attendants—Infirmary Medical Officers and Active Service—The Salaries of Infirmary Assistant Medical Officers—What to Omit in Your Applications for Promotion—Winsley Sanatorium Administration Scheme—The Management of Sanatorium Patients—The Director of the Medical Service of the Welsh Army Corps—The Welsh War Hospital, Netley—Sanatorium Benefit and the Poor Rate—Death of a Hospital Architect—The Infirmary's Relation to the Mental Hospital—A Bishop on Medical Missions—The New Stationery Office as a Hospital—Illustrated Appeals—"Glamorgan and Monmouth Hospital for the French Army"—The Poor-Law Standard and Infirmary Training Schools—An Invalid Bequest to a Hospital—The Employment of Belgian Pharmacists—This Week's Drug Market—To Our Readers.

**Some Smaller Hospitals and the Wounded** ... 194

PAGE

**Surgical Experiences in the Present War** ... 193

"Really Heroic Work."
The Unequalled Efficiency of the R.A.M.C.

**Sidelights upon the War** ... ... ... ... 195

Reflections and Reminiscences.

**The London Panel Committee** ... ... ... 196

**The Royal Infirmary, Glasgow** ... ... ... 197

II.—Some Special Features of Importance.
The New Pathological Building at Glasgow Royal Infirmary.

**Hospitals and the War** ... ... ... ... 203

**Work for the Wounded of the Allied Armies**

Cambridge: 1,250 Beds Nearly Completed—Chatham District: The Cost of Temporary Hospitals—Leeds: A Soldier's View of Civilian Hospitals—Leicester: 5th Northern General Hospital—Salisbury: New Soldiers' Ward at the Infirmary—St. Leonards New General Hospital—Bristol: New Southmead Infirmary—Bristol—Sheffield: What the Base Hospital has Done.

**The Editor's Letter-Box** ... ... ... ... 205

The War and Nervous Diseases—Hospital Statistics—Discipline in Convalescent Homes.

**Round the World** ... ... ... ... ... 208

Fellow-Workers and the Roll of Honour.

**The Institutional Worker** ... ... (Suppl.) 1

Padded Rooms.

